# Remarkable features of ovarian morphology and reproductive hormones in insulin-resistant Zucker fatty (fa/fa) rats

**DOI:** 10.1186/1477-7827-8-73

**Published:** 2010-06-24

**Authors:** Hiroyuki Honnma, Toshiaki Endo, Tamotsu Kiya, Ayumi Shimizu, Kunihiko Nagasawa, Tsuyoshi Baba, Takashi Fujimoto, Hirofumi Henmi, Yoshimitsu Kitajima, Kengo Manase, Shinichi Ishioka, Eiki Ito, Tsuyoshi Saito

**Affiliations:** 1 Department of Obstetrics and Gynecology, Sapporo Medical University School of Medicine, South 1 West 16, Chuo-ku, Sapporo 060-8543, Japan

## Abstract

**Background:**

Zucker fatty (fa/fa) rats are a well-understood model of obesity and hyperinsulinemia. It is now thought that obesity/hyperinsulinemia is an important cause of endocrinological abnormality, but to date there have been no reports on the changes in ovarian morphology or the ovarian androgen profile in rat models of obesity and insulin resistance.

**Methods:**

In this study we investigated the effects of obesity and hyperinsulinemia on ovarian morphology and the hormone profile in insulin-resistant Zucker fatty rats (5, 8, 12 and 16 weeks of age, n = 6-7).

**Results:**

Ovaries from 5-week-old fatty rats had significantly greater total and atretic follicle numbers, and higher atretic-to-total follicle ratios than those from lean rats. Ovaries from 12- and 16-week-old fatty rats showed interstitial cell hyperplasia and numerous cysts with features of advanced follicular atresia. In addition, serum testosterone and androstenedione levels significantly declined in fatty rats from age 8 to 16 weeks, so that fatty rats showed significantly lower levels of serum testosterone (12 and 16 weeks) and androstenedione (all weeks) than lean rats. This may reflect a reduction of androgen synthesis during follicular atresia. Serum adiponectin levels were high in immature fatty rats, and although the levels declined significantly as they matured, it remained significantly higher in fatty rats than in lean rats. On the other hand, levels of ovarian adiponectin and its receptors were significantly lower in mature fatty rats than in lean mature rats or immature fatty rats.

**Conclusions:**

Our findings indicate that ovarian morphology and hormone profiles are significantly altered by the continuous insulin resistance in Zucker fatty rats. Simultaneously, abrupt reductions in serum and ovarian adiponectin also likely contribute to the infertility seen in fatty rats.

## Background

Zucker fatty (fa/fa) rats are a well-understood model of obesity and hyperinsulinemia, which arise when the animals are about 4 weeks of age and gradually worsen thereafter [1]. The cause of the obesity is overeating induced by a loss of leptin signaling associated with a leptin receptor mutation. Zucker fatty rats also have several endocrine and metabolic abnormalities, including a low metabolic rate [2], hyperinsulinemia [3,4], abnormal estrous cyclicity [5] and infertility [3]. They are not diabetic, however. It is now thought that their obesity/hyperinsulinemia is an important cause of their endocrinological abnormality, but to date there have been no reports on the changes in ovarian morphology or the ovarian androgen profile in rat models of obesity and insulin resistance.

Adiponectin is a 30-kDa protein comprised of 247 amino acids in four domains and has been shown to possess insulin-sensitizing [6-8], anti-atherogenic [9,10] and anti-inflammatory properties [11]. It also reportedly inhibits apoptosis among endothelial cells [12]. Zucker fatty rats show higher serum adiponectin level than lean rats [13]. Adiponectin and its receptors are expressed in rat testis [14] and ovary [15,16] and play a significant role in steroidogenesis [17], but the mechanism underlying the changes in ovarian adiponectin expression caused by obesity/insulin resistance remains unknown. In an earlier study, we reported that by injecting immature rats with androgen (dehydroepiandrosterone), an animal model of polycystic ovary could be produced that exhibits the salient features of human PCOS - i.e., prepubertal female rats develop cysts and become anovulatory and uncyclic [18], and the follicles undergo atresia or exhibit various stages of cystogenesis up to complete pathological transformation into a follicular cyst [19,20]. Our aim in the present study, therefore, was to investigate the ovarian morphology and hormone profile in Zucker fatty rats, focusing on serum androgen levels and ovarian adiponectin expression.

## Methods

### Animal treatments and tissue collection

Female Zucker fatty rats (Slc:Zucker-fa/fa) and lean rats (Slc:Zucker-+/+) (4, 7, 11 and 15 weeks of age) were purchased from Japan SLC (Shizuoka, Japan). Vaginal smears were collected each morning by lavage for about 2 weeks to determine estrous cyclicity [5]. Rats were usually sacrificed by decapitation on day 4 of their estrous cycle, in diestrus during day, and after 12 h of fasting (5, 8, 12 and 16 weeks of age). The ovaries were then dissected out, trimmed, weighed, flash-frozen on dry ice, and stored at -80°C until use. Animals used in this study were maintained in accordance with the guidelines of the Animal Resources Center of the Sapporo Medical University School of Medicine.

### RIAs

Serum FSH, LH, estradiol, testosterone and androstenedione levels were determined using RIA kits (DPC Co., Los Angeles, CA) as previously described [21]. Serum adiponectin was measured using a commercially available enzyme-linked immunosorbent assay kit (Otuska Pharmaceuticals Co., Japan).

### TdT-mediated dUTP-biotin nick end-labeling (TUNEL)

TUNEL was carried out using an ApopTag Plus Peroxidase In Situ Apoptosis Detection Kit (Intergen, NY) as described previously [22]. Tissue samples embedded in paraffin were cut into 5-μm-thick sections, which were then deparaffinized in xylene and rehydrated through a graded ethanol series in distilled water. Each slide was first incubated with proteinase K, after which endogenous peroxidase activity was removed by treatment with 0.3% H_2_O_2 _for 20 min at room temperature. After intense washing with distilled water for 15 min, the sections were soaked in TdT buffer for 15 min and then incubated for 60 min at room temperature in TdT buffer containing biotin. The biotinylated dUTP incorporated into the nuclear DNA was reacted with HRP-conjugated streptavidin (1:100 dilution) for 30 min at room temperature, after which the color was developed by immersing the sections for 10 min into 50 mmol/l Tris-HCl buffer (pH 7.6) containing 0.3 mg/ml DAB, 10 mmol/l imidazole and 0.003% H_2_O_2_. Finally, the sections were counterstained with hematoxylin. Negative controls were reacted in parallel with the omission of the terminal transferase.

### Real-time RT-PCR

Real-time RT-PCR analysis was carried out as described previously [23]. Briefly, PCR was carried out using a DyNAmo HS SYBR Green qPCR kit (Finnzymes, Espoo, Finland), and the fluorescent signals were detected using an ABI PRISM 7700 sequence detection system (Applied Biosystems, Foster City, CA, USA). The oligonucleotide sequences of the primers used were described in Table 1. The thermal cycling protocol entailed an initial denaturation step at 95°C for 15 min and 40 cycles at 94°C for 10 s, 60°C for 20 s, and 72°C for 30 s. There is no significant difference in the amount of beta-actin mRNA from total RNA extract between 5 week and 16 week ovary after same extraction procedure. Experiments were carried out in triplicate, and the levels of adiponectin and its receptors were normalized to those of rat beta-actin.

**Table 1:** Rat oligonucleotide primer sequences for RT-PCR

Primer	Sequence	**Product size Accession No**.
Adiponectin		
Forward	5'-gcccagtcatgaagggatta-3'	188 bp
Reverse	5'-gaccaagaacacctgcgtct-3	NM_144744
AdipoR1		
Forward	5'-cttctactgctccccacagc-3'	139 bp
Reverse	5'-tcccaggaacactcctgctc-3	NM_207587
AdipoR2		
Forward	5'-tgggaagttttgttccttgg-3'	201 bp
Reverse	5'-gcaaggtagggatgattcca-3	NM_001037979
Beta-actin		
Forward	5'-aggcccctctgaaccctaag-3'	195 bp
Reverse	5'-ggagcgcgtaaccctcatag-3	NM_031144

### Western blot analysis

Western blot analysis was carried out as described previously [23]. Ovaries were homogenized (100 mg wet weight/ml) in ice cold PBS (10 mM sodium phosphate and 150 mM sodium chloride, pH 7.8) containing 0.2% Triton X-100 using a Teflon glass tissue grinder (15 strokes), after which the homogenates were centrifuged (12,000 × *g *at 4°C for 20 min), and the supernatant fractions were collected for protein assay. Samples of ovarian extract containing 20 μg of protein were then separated by 12.5% SDS-PAGE under reducing conditions and transferred to nitrocellulose membranes using an electroblotting apparatus. Nonspecific binding sites were blocked by incubating the membrane overnight at room temperature in PBS containing 0.05% Tween 20 (TPBS) and 5% skim milk. After five washes for 5 min each in TPBS, the membrane was incubated with the primary antibody (diluted 1:5000 with PBS-BSA for rat adiponectin) for 90 min at room temperature in a humidified chamber. A rat anti-adiponectin antibody was obtained from Abcam (Cambridge, UK). The membrane was then washed again five times for 5 min each in TPBS, incubated with anti-rabbit HRP conjugate (1:5000 dilution) for 50 min at room temperature in a humidified chamber, and finally washed five times for 5 min each in TPBS. For visualization, the blots were incubated with enhanced chemiluminescence reagent (Amersham Pharmacia Biotech UK Ltd., Buckinghamshire, UK) for 1 min and exposed to x-ray film for 15 s in a dark room. The densities of the resultant bands were measured using NIH Image (version 1.61; NIH, Bethesda, MD).

### Immunohistochemical analysis

Immunohistochemical analysis was carried out as described previously [22]. Tissues embedded in paraffin were cut into 5-μm-thick sections and mounted on APS-coated glass slides. The slides were then deparaffinized in xylene and placed on a hotplate at 90°C, covered with STUF-solution (Serotec Ltd, UK) for 10 min, rinsed several times with PBS, and incubated in 0.6% H_2_O_2 _in methanol for 30 min at room temperature to eliminate endogenous peroxidase activity. The sections were then blocked in Block Ace (Dainippon Pharmaceutical Co, Japan) for 30 min at room temperature, after which they were incubated at room temperature, first with primary antibody (diluted 1:100 with PBS-BSA for adiponectin) for 60 min and then with biotinylated secondary antibody for 30 min. We visualized the labeling using the ABC method (Vectastaign ABC Elite kit; Vector Laboratories, USA) with DAB as a substrate and hematoxylin as a counterstain. As a negative control, some slides were stained with hematoxylin and eosin (HE); alternatively normal rabbit serum was used instead of the primary antibody, or the primary antibody was omitted altogether (data not shown).

### Electron microscopy

Isolated ovaries were fixed for 2 h in 2.5% glutaraldehyde buffered with sodium cacodylate buffer (0.1 M, pH 7.4) and post-fixed for 2 h in 1.0% osmium tetroxide buffered with the same buffer. After dehydration in a graded ethanol series, specimens were placed in Epon 812, cut into semi-thin sections (0.3-0.5 μm) and stained with toluidine blue for light microscopy. Ultrathin sections were then cut, stained using uranyl acetate and lead citrate, and examined in an electron microscope at 125 kV (H-700, Hitachi Japan).

### Statistics

**Figure 1:**
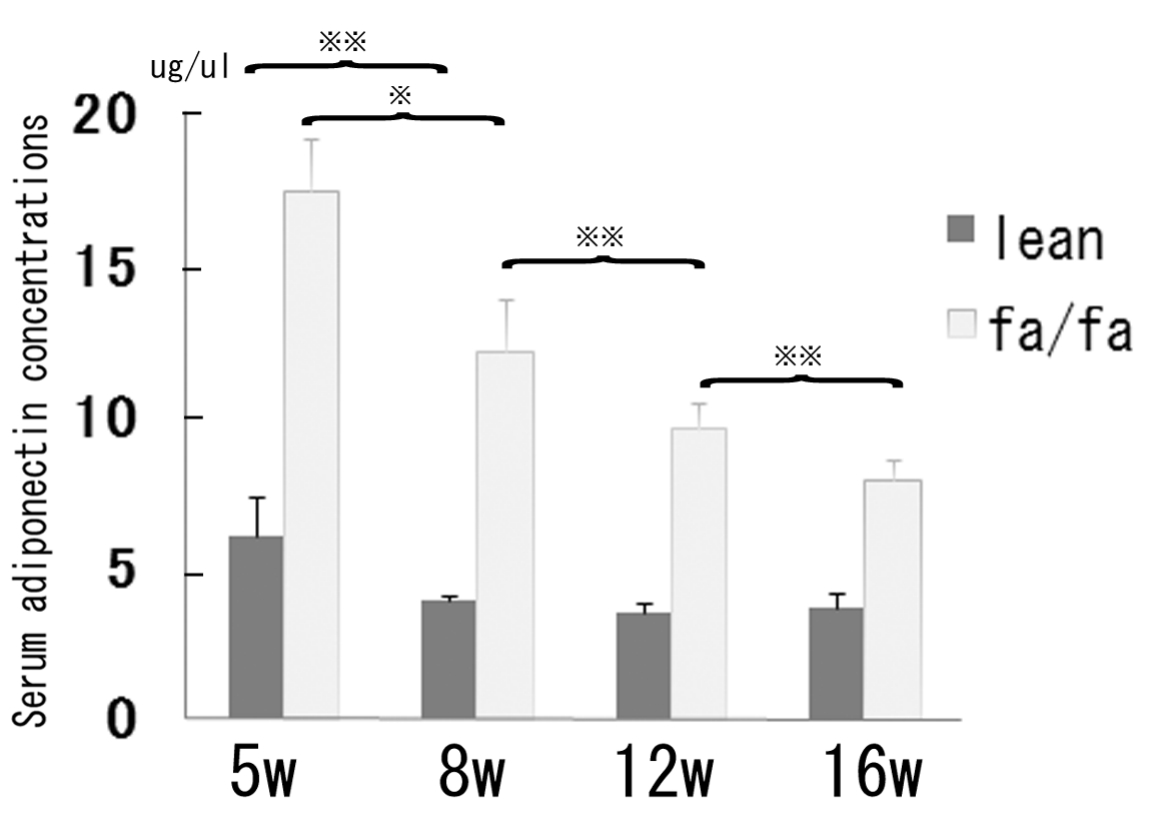
**Serum adiponectin expression in Zucker fa/fa and lean rats**. Serum adiponectin levels were measured in fatty and lean rats at the indicated ages in diestrus during day after fasting the rats for 12 h (n = 6 in each group). Values are means ± SD. (※, P < 0.01; ※※, P < 0.05)

**Figure 2:**
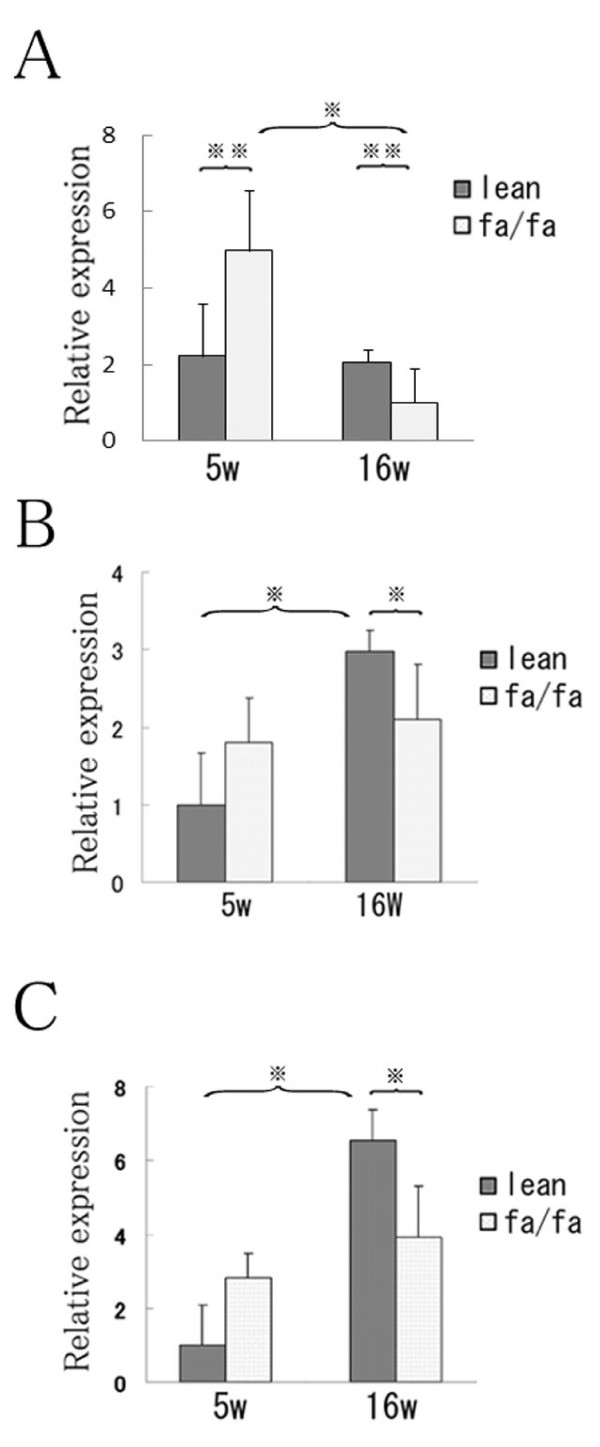
**Ovarian expression of adiponectin, AdipoR1 and AdipoR2 mRNA in Zucker fa/fa and lean rats**.The oligonucleotide sequences of the primers used were described in Table 1. The thermal cycling protocol entailed an initial denaturation step at 95°C for 15 min and 40 cycles at 94°C for 10 s, 60°C for 20 s, and 72°C for 30 s. There is no significant difference in the amount of beta-actin mRNA from total RNA extract between 5 week and 16 week ovary after same extraction procedure. Relative expression of adiponectin (A) adipoR1(B) and AdipoR2(C) mRNA normalized to the level of rat β-actin mRNA. Values are means ± SD; n = 4 in each group; ※, P < 0.01; ※※, P < 0.05.

**Figure 3:**
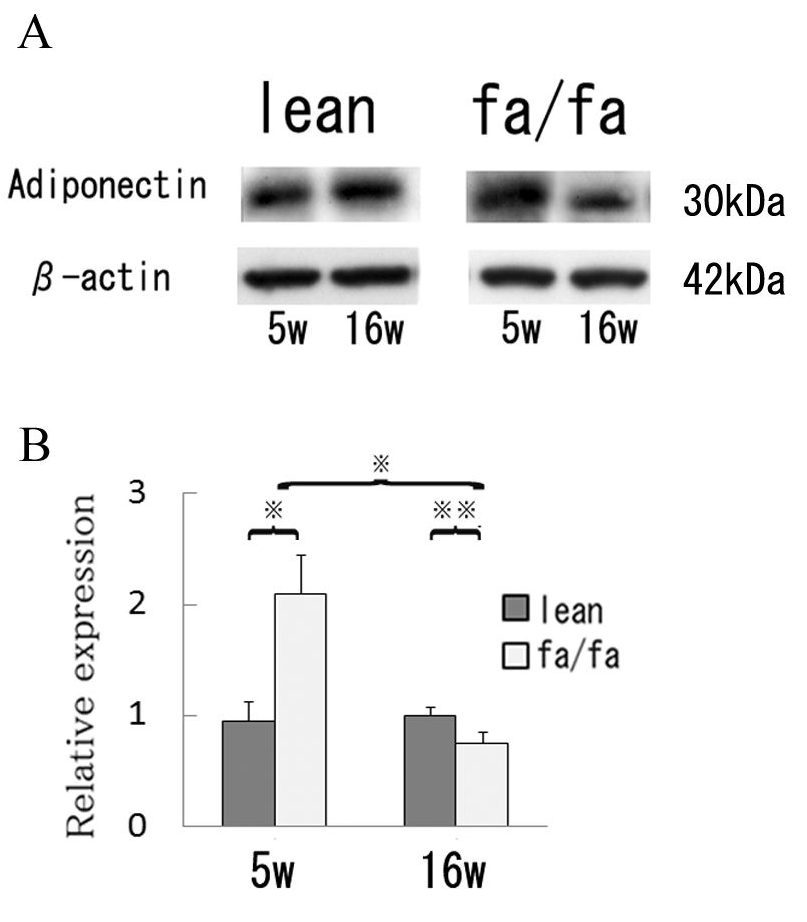
**Western blot analysis of ovarian expression of adiponectin protein in Zucker fa/fa and lean rats**.A) Samples (20 μg of protein) of ovarian tissue extract were subjected to SDS-PAGE, transferred to nitrocellulose membranes, and probed with rat anti-adiponectin antibody. B) Band densities. Values are means ± SD; n = 4 in each group; ※, P < 0.01; ※※, P < 0.05.

Quantitative data are presented as means ± SD. For these experiments, we used two groups and made measurements at several different time points. We compared the groups at the different time points using two-factor factorial ANOVA with a post-hoc Scheffe test (Fig. 1, Fig. 2, Fig. 3 and Table 2), or we did a point-by-point comparison between the two groups using Mann-Whitney's U test (Fig 5). Values of P < 0.05 were considered significant.

**Figure 4:**
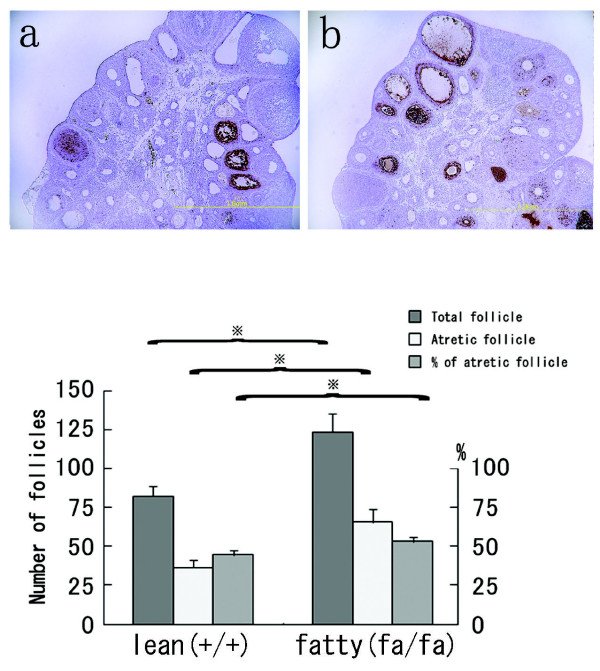
**Apoptotic follicles in Zucker fa/fa and lean rats at 5 weeks of age**. *Upper*: TUNEL analysis of ovaries from lean (a) and fa/fa (b) rats. TUNEL-positive cells were scattered among the granulosa cells, mainly in atretic follicles (magnification, 40×). *Lower*: Total and atretic follicle numbers and the percentages of follicles deemed to be atretic (n = 6 in each). Values are means ± SD. (※, P < 0.01)

**Figure 5:**
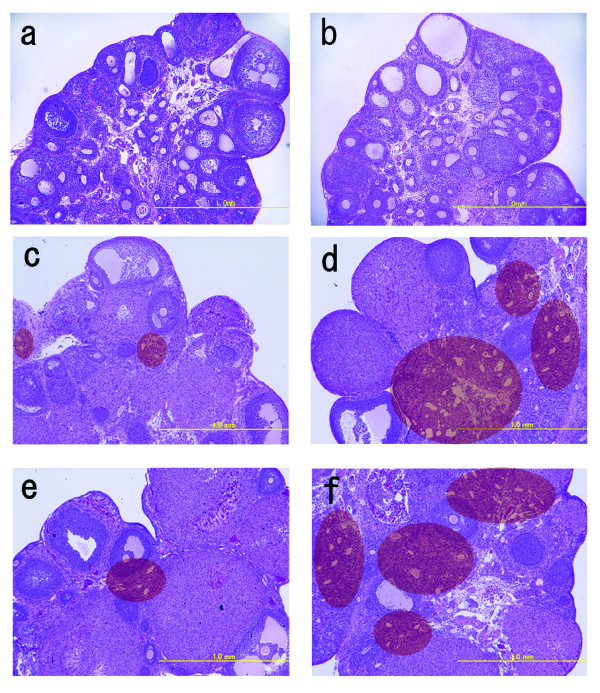
**Ovarian morphology in Zucker fa/fa and lean rats**.Sections are representative examples of HE-stained ovaries: a) lean rat at 5 weeks of age; b) fa/fa rat at 5 weeks of age; c) lean rat at 12 weeks of age; d) fa/fa rat at 12 weeks of age; e) lean rat at 16 weeks of age; f) fa/fa rat at 16 weeks of age. The areas within the red circle show interstitial cell hyperplasia and multiple cysts. (Magnification, 40×).

**Table 2:** Summary of body weights, estrous cycle and hormonal data for Zucker fa/fa and lean rats

		5 weeks of age	8 weeks of age	12 weeks of age	16 weeks of age
*Body weight (g)*	lean	80.32 +/- 5.80^c^	128.15 +/- 5.30^c^	151.61 +/- 8.83^c^	171.39 +/- 11.34
	fa/fa	108.13 +/- 6.78^a,c^	224.36 +/- 4.46^a,c^	282.10 +/- 14.61^a,c^	340.85 +/- 18.38^a^
*% of irregular estrous cycle*	lean	ND	0 (0/6)	0 (0/6)	0 (0/6)
	fa/fa	ND	14.3%(0/6)	28.6% (0/6)	71.4% (0/6)
*FSH (ng/ml)*	lean	ND	5.07 +/- 0.06	3.01 +/- 0.22	4.63 +/- 0.65
	fa/fa	ND	5.15 +/- 0.21	2.87 +/- 0.45	3.81 +/- 0.74
*LH (ng/ml)*	lean	ND	0.97 +/- 0.23	1.10 +/- 0.14	1.03 +/- 0.18
	fa/fa	ND	1.15 +/- 0.57	1.57 +/- 0.18	1.21 +/- 0.23
*Estradiol (pg/ml)*	lean	ND	13.26 +/- 1.57^d^	24.45 +/- 1.41^c^	65.51 +/- 18.07
	fa/fa	ND	8.53 +/- 0.29^b,d^	14.38 +/- 0.98^a^	17.70 +/- 1.47^a^
*Testosterone (ng/ml)*	lean	0.16 +/- 0.04^c^	0.45 +/- 0.02	0.44 +/- 0.08	0.35 +/- 0.16
	fa/fa	0.07 +/- 0.01^c^	0.34 +/- 0.12^d^	0.28 +/- 0.14^b,d^	0.16 +/- 0.05^b^
*Androstendione (ng/ml)*	lean	0.70 +/- 0.35^c^	1.50 +/- 0.46	1.55 +/- 0.52	1.35 +/- 0.52
	fa/fa	0.13 +/- 0.06^b,c^	0.65 +/- 0.12^b,d^	0.53 +/- 0.22^a,c^	0.33 +/- 0.15^a^

Rats were usually sacrificed by decapitation on day 4 of their estrous cycle, in diestrus during day, and after 12 h of fasting (5, 8, 12 and 16 weeks of age). Data are presented as means ± SD; n = 6 or 7 in each group. a: P < 0.01, b: P < 0.05 vs. lean rats, c: P < 0.01, d: P < 0.05 vs. later weeks, ND: not done.

## Results

### Estrous cyclicity

All lean rats had normal 4-day estrous cycles. On the other hand, the estrous cycles of Zucker fatty rats showed increasing irregularity as the rats aged (Table 2), and their diestrous periods were prolonged (data not shown). For example, at 8 weeks of age only one of the seven rats tested showed abnormal estrous cyclicity, but at 16 weeks five of the seven rats showed abnormal estrous cyclicity. Thus the incidence of abnormal cyclicity increased in fatty rats as obesity/insulin resistance became more severe.

### Body weight

In all groups, the fatty rats were significantly heavier than the lean rats (Table 2).

### Hormone profile in Zucker fa/fay and lean rats

Hormone assays were carried using serum samples collected from rats at 5, 8, 12 and 16 weeks of age, during diestrous, after they were fasted for 12 h (n = 6 or 7 in each group). In lean rats, serum testosterone increased significantly as they matured during the period from age 5 to 8 weeks, after which the levels remained stable until the rats were 16 weeks of age. In fatty rats, by contrast, serum testosterone significantly declined during the period from age 8 to 16 weeks, and was significantly lower in fatty rats than in lean rats at age 12 and 16 weeks. The serum androstenedione profile was similar to the serum testosterone profile in lean and fatty rats, though serum androstenedione levels were significantly higher in lean rats than fatty rats at all times. In lean rats, serum estradiol levels significantly increased as the rats matured, but in fatty rats estradiol levels remained stable and were significantly lower than in lean rats at all times. There were no differences in FSH or LH levels between fatty and lean rats at any age (Table 2). In addition, although serum adiponectin levels were significantly higher in fatty rats than lean rats, they declined abruptly in fatty rats during the period from age 5 to 8 weeks (Fig. 1).

### Expression of adiponectin in ovaries of Zucker fa/fa and lean rats

In 5-week-old rats, ovarian expression of adiponectin mRNA and protein corresponded to the levels of adiponectin in serum; that was not the case at 16 weeks, however. Although ovarian levels of adiponectin mRNA (Fig. 2A) and protein (Fig. 3) were significantly higher in fatty rats than in lean rats at 5 weeks of age, the levels significantly declined in fatty rats as they matured from age 5 to 16 weeks. In lean rats, by contrast, there were no age-dependent changes in adiponectin expression. Consequently, expression of adiponectin was significantly lower in fatty rats than lean rats at 16 weeks.

### Expression of adipoR1/R2 in ovaries of Zucker fa/fa and lean rats

The expression patterns of adipoR1 and adipoR2 mRNA were similar. Expression of AdipoR1/R2 mRNA was very low in lean rats at 5 weeks of age, but was significantly (P < 0.01) higher at 16 weeks than at 5 weeks (Fig. 2B, C). By contrast, levels of adipoR1/R2 mRNA expression did not significantly differ between 5- and 16-week-old fatty rats. Moreover, expression of adipoR1/R2 mRNA was significantly (P < 0.05) lower in fatty rats than lean rats at 16 weeks of age.

### Ovarian morphology in Zucker fa/fa and lean rats

At 5 weeks of age, the ovaries of fatty rats contained significantly (P < 0.01) larger numbers of antral/preantral follicles than those of lean rats (Fig 4a, b). This prompted us to carry out a TUNEL analysis of the follicles and classify them as atretic if they had five or more apoptotic granulosa cells [24]. We found TUNEL-positive cells to be scattered among the granulosa cells within atretic follicles in both fatty and lean rats; however, the incidence of TUNEL-positivity was significantly higher in the antral/preantral follicles of the fatty rats (Fig. 4). Fatty rats also had slightly greater numbers of healthy follicles than lean rats at this age.

**Figure 6:**
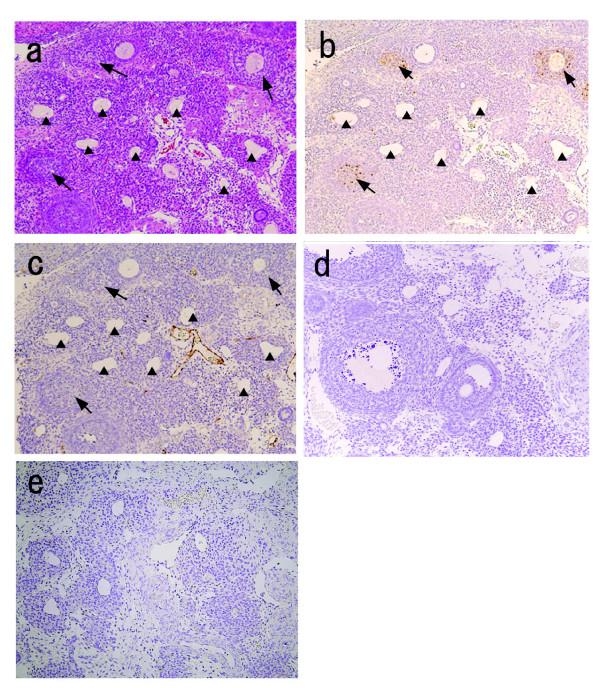
**Representative sections showing ovaries from mature Zucker fa/fa rats with multiple cysts**. a) HE staining. b) TUNEL. c) Immunohistochemical staining of anti-factor VIII. d) negative control (TUNEL). e) negative control (anti-factor VIII). Arrows show TUNEL-positive atretic follicles; arrowheads show cysts within interstitial cells. Factor VIII staining was seen only within vessels (magnification, 100×).

**Figure 7:**
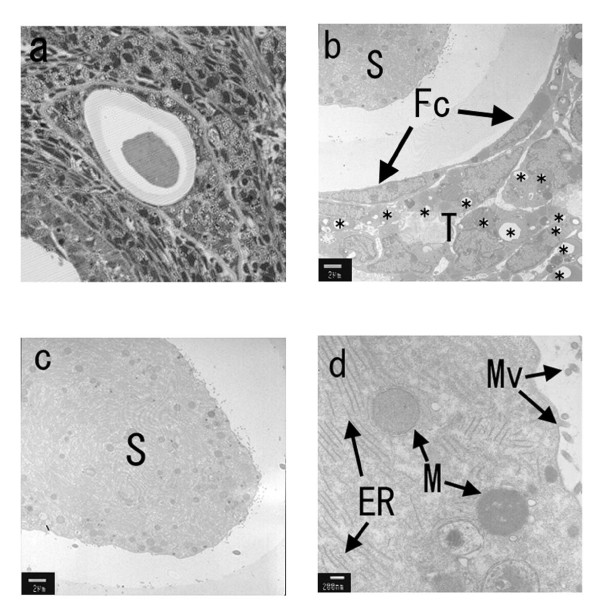
**Electron microscopic analysis of a representative cyst in an ovary from a mature fa/fa rat**. a) Photomicrograph of the cyst stained with toluidine blue (Magnification, 100×). b) Electron micrograph of the cyst (magnification, 100×; bar, 2 μm). The region bordering the cyst was comprised of one layer of fibrous cells in addition to theca cells that contained fat deposits. No normal granulosa cells were seen. c) Electron micrograph showing the interior of the cyst (magnification, 2,500×; bar, 2 μm). A possible degenerating oocyte is seen. d) Higher magnification electron micrograph of the homogenous substance showing mitochondria, endoplasmic reticulum and microvilli: S, homogeneous substance; Fc, fiver cell; T, theca cell; *, fat deposit; Mv, microvilli; M, mitochondria; ER, endoplasmic reticulum (magnification, 20,000×; bar, 200 nm).

At 12 and 16 weeks of age, a few corpora lutea and antral/preantral follicles were seen in ovaries from fatty rats, but the interstitial tissue appeared to be enlarged and to contain numerous cysts (Fig. 5d, f). Similar cysts were seldom seen in the ovaries of lean rats (Fig. 5c, e). Figure 6a shows a cyst present in the ovary of a 16-week-old fatty rat. These cysts were negative for both TUNEL and factor VIII staining (Fig. 6), indicating there were no blood vessels or apoptotic cells present. Electron microscopic findings showed the cysts to be composed of one layer of fibrous cells (Fc), outside of which were numerous theca cells (T) containing fat deposits (Fig. 7b). No normal granulosa cells and no zona pellucida were seen, but there was a large amount of homogenous substance (S) within the cysts (Fig. 7c). On the other hand, examination of the homogenous substance at higher magnification revealed the presence of endoplasmic reticulum (ER), mitochondria (M) and microvilli (Mv), which might have comprised an oocyte at one time (Fig 7d). These findings indicate that the numerous cysts may be the final manifestations of atretic follicles in mature fatty rats.

## Discussion

In the present study, we found that ovarian morphology and hormone profiles significantly differed between Zucker fatty and lean rats. As the fatty rats matured and became more obese, they showed follicular atresia, reduced steroidogenesis, abnormal estrous cyclicity and abrupt changes in adiponectin levels. Ovaries from immature (5 weeks of age) fatty rats showed significantly greater total and atretic follicle numbers than ovaries from immature lean rats. By 8 weeks of age estrous cyclicity was abnormal in one of seven fatty rats, and by maturity (12 or 16 weeks of age) ovaries from fatty rats showed attenuated follicle growth and multiple cysts within the interstitial tissue. In addition, the incidence of abnormal estrous cyclicity gradually increased from 28.6% at 12 weeks of age to 71.4% at 16 weeks of age. Immunohistochemical analysis of the cysts indicated they were neither microvessels nor TUNEL-positive atretic follicles.

Devine et al. reported that in Fisher 344 rats, granulosa cells are not seen in atretic follicles, and that the degenerating oocyte becomes segmented, though the zona pellucida remains intact throughout the entire process and continues to enclose the oocyte segments [25]. Our electron microscopic analysis revealed that granulosa cells and the zona pellucida were not present within the cysts in fatty rats, but that they did contain endoplasmic reticulum, mitochondria and microvilli, which might have comprised an oocyte at one time. Despite our negative immunohistochemical and TUNEL findings, this suggests these cysts are follicles at an advanced stage of atresia. It may be that follicular atresia is accelerated in immature fatty rats, which would account for the numerous atretic follicles seen. The atretic follicles in turn become the cysts seen in mature rats. Of course, the morphological changes described above must be associated with alterations in ovarian steroidogenesis. In lean rats, androgen levels significantly increased during the period from age 5 to 8 weeks, after which levels remained stable until 16 weeks. In fatty rats, by contrast, androgen levels significantly declined during the period from age 12 to 16 weeks, during which time follicular atresia progressed. We suggest that the low levels of androgens may reflect the reduction in the numbers of healthy follicles, or androgen synthesis may have declined, even though the number of follicles did not change during the process of follicular atresia. The low androgen levels also might have inhibited follicle growth.

A variety of molecules (glucose and fatty acids, among others) are thought to be directly involved in the regulation of fertility, acting through fuel sensors at every level of the hypothalamo-pituitary-gonadal axis [26,27]. Our present findings suggest adiponectin may be one of those molecules. We had initially expected that obesity-induced insulin resistance/hyperinsulinemia would stimulate androgen production in Zucker fatty rats, as is seen in polycystic ovary syndrome. Surprisingly, however, serum testosterone and androstenedione significantly declined in fatty rats during the period from age 8 to 16 weeks, though levels remained nearly stable and higher (as compared to fatty rats) in lean rats at the same age. Although it was not proved in this study that adiponectin affects androgen synthesis, Lagaly et al. showed that adiponectin reduces insulin-induced androstenedione production in theca cells [17]. Moreover, it has been proposed in several reports that there is a negative correlation between serum androgen [28,29] or estradiol [30] levels and serum adiponectin levels under certain circumstances. It is thus plausible that androgen synthesis is affected by the excessively high serum adiponectin levels seen in immature fatty rats. We therefore suggest that continuously high serum adiponectin levels prior to vaginal opening might downregulate ovarian steroidogenesis. Alternatively, the low ovarian steroidogenesis may contribute to the accelerated follicle atresia see in fatty rats.

In the estrous cycle, circulating reproductive hormones in fatty and lean rats were previously investigated by Whitaker et al.. They reported about plasma estradiol concentration and mentioned that the pattern of estradiol over the estrous cycle was similar in both rats and small difference was found in diestrous, although they did not analyze the statistical significance between fatty and lean rats [31]. In another study they also reported serum FSH, LH, progesterone and prolactin concentrations in fatty rats; proestrous peak concentration of LH was reduced and that of FSH was similar [32]. Above data agree to our hormonal data about FSH, LH and estradiol in diestrous. In addition, they reported progesterone and prolactin concentrations were higher during most of the estrous cycle in fatty rats [32]. Unfortunately, we did not investigate hormonal data at each estrous cycle in this study. However, further studies are needed to clarify complete hormonal status of these rats.

In mature Zucker fatty rats, an abrupt reduction in serum adiponectin appeared to be correlated with acceleration of follicular atresia. Adiponectin is reportedly expressed mainly in theca cells, granulosa cells, oocytes, and corpora lutea in rat ovaries [14,17]. We found that ovarian expression of adiponectin mRNA and protein was significantly lower in mature fatty rats than immature fatty rats. Moreover, mature fatty rats showed significantly lower ovarian adiponectin levels than lean rats, which is contrary to the situation in serum, where adiponectin levels are higher in fatty rats. This inconsistency between serum and ovarian adiponectin levels in rats has been observed previously [15], though the underlying mechanism remains unclear. We also observed that in mature fatty rats, the increase in ovarian AdipoR1/R2 expression is significantly attenuated, as compared to lean rats. Adiponectin also plays a role in determining oocyte quality [33,34] and, as mentioned above, alterations in the ovarian expression of adiponectin and its receptor system could affect ovarian steroidogenesis in granulosa and theca cells in fatty rats. Collectively then, our findings suggest the reduction in ovarian steroidogenesis is related to the changes in ovarian morphology seen in Zucker fatty rats, including the reductions in the numbers of healthy follicles and in ovarian adiponectin production.

## Conclusions

We have shown that ovarian morphology and androgen profiles differ substantially between Zucker fatty and lean rats, and that the reproductive abnormalities seen in the fatty rats gradually worsen as the rats mature. As obesity/insulin resistance became more severe in the fatty rats, androgen levels declined and follicular atresia accelerated. This was accompanied by dramatically altered adiponectin levels and a higher incidence of abnormal cyclicity. The precise effects of these adiponectin alterations on ovarian function, and thus the cause of infertility in Zucker fatty rats, remain to be determined.

## Competing interests

The authors declare that they have no competing interests.

## Authors' contributions

TE is the corresponding author. AS and SI carried out the Western blot analysis. NK carried out Real-time RT-PCR analysis. TK and HH participated in the design of the study. TB performed the statistical analysis. YK and KM carried out animal treatment and tissue collection. EI carried out the Electron microscopy. TS participated in study design and helped to draft the manuscript. All authors read and approved the final manuscript.
